# Branched DNA-Based Electrochemical Biosensor for Sensitive Nucleic Acids Analysis with Gold Nanoparticles as Amplifier

**DOI:** 10.3390/ijms241612565

**Published:** 2023-08-08

**Authors:** Zhikun Zhang, Chunyan Shang, Cuixia Hu, Yumin Liu, Jilong Han

**Affiliations:** School of Chemical and Pharmaceutical Engineering, Hebei University of Science and Technology, Shijiazhuang 050018, China

**Keywords:** amplifier, branched DNA, diagnosis, detection, nucleic acids

## Abstract

A branched DNA-based electrochemical biosensor was designed to sensitively detect specific nucleic acids. On this platform, novel a branched DNA with three sticky ends could be used as a biosensor to sensitively and specifically detect nucleic acids. Meanwhile, we also employed branched DNA-modified AuNPs as a signal amplifier to further improve the sensitivity. Branched DNA sensors, target DNA, and DNA-modified AuNPs formed a sandwich structure to produce an electronic signal for target DNA detection. The reaction primarily involved DNA hybridization without bulky thermal cyclers and enzymes. We proved that the hybridization reaction easily occurred under different conditions, such as the NaCl concentration, reaction time, pH, and temperature, except for a pH lower than 4. The limit of detection could go as low as 0.09 pM (S/N = 3) with excellent specificity and selectivity. There was a correlation curve relationship between the peak current and the logarithm of the target DNA concentration (0.10 pM to 10 nM). The correlation coefficient reached 0.987. The electrochemical platform enables a branched DNA nanostructure to determine nucleic acids for disease diagnosis.

## 1. Introduction

Nowadays, irregular concentrations of particular nucleic acids in the human body are more closely associated with the occurrence and progress of different tumors and other pathological conditions [1,2]. Therefore, particular nucleic acids could be employed as biomarkers in early-stage tests of disease diagnosis [3,4]. The detection platform with easy operation for self-diagnosis could effectively improve the survival and curative ratio of patients in resource-limited settings, particularly those with tumors or cancers. Traditional numerical strategies had been built to detect nucleic acids with high specificity and sensitivity, such as northern blotting [5], DNA microarrays [6], and quantitative RT-PCR (qRTPCR) [7,8]. However, those strategies generally require bulky thermal cycles, enzyme involvement, costly equipment, complex operation, time, and technical personnel. The drawbacks meant it was impossible for these technologies to satisfy the public needs in resource-limited areas for disease self-diagnostics. In order to overcome those defects, new strategies had been developed to improve the performance of nucleic acid detection systems for Point-of-Care testing, such as fluorescence [9], colorimetry [10], electrochemistry [11], and DNA nanomaterials [12]. Due to their sensitivity and simple operation, electrochemical biosensors are becoming increasingly important as signal detectors to detect nucleic acids, with their rapid response and low cost, in several fields such as food safety [13], environmental [14], and diagnosis [15]. The sensitive detection of specific nucleic acids was challenging for nucleic acid-based diseases because of the low abundance of nucleic acids in the real specimen. As for nucleic acid detection, the key to solving this problem lies in amplification strategies, which could amplify the signal response. So far, numerous technologies for nucleic acid amplification have been proposed to develop a platform for nucleic acid determination, such as loop-mediated isothermal amplification (LAMP) [16,17], rolling circle amplification (RCA) [18], the ligase chain reaction (LCR) [19], and nucleic acid sequence-based amplification (NASBA) [20]. However, the above systems suffer from strict conditions for enzyme storage and catalytic conditions such as environmental temperature and pH, making these systems not ideal for convenient detection. Thus, detection strategies free from enzymes are attracting increasing attention.

DNA nanomaterials possess unique molecular programmability and nanoscale controllability, which have been extensively employed in molecular detection [21,22,23]. Isothermal detection systems based on DNA assembly do not need an enzyme with a rapid reaction. DNA nanotechnology has opened up new avenues for nucleic acid-based diagnostics [22,24]. However, it is still urgently needed to further increase the sensitivity via simple operation without enzymes for Point-of-Care testing in disease diagnostics. Herein, we designed branched DNA as a probe and DNA-modified AuNPs as an amplifier to construct a nucleic acid detection platform, which did not require any enzymes, via Watson–Crick base pairing in isothermal conditions [25]. In this system, branched DNA was first modified on the surface of the electrode realizing the recognition, and DNA-modified AuNPs simultaneously amplified the target nucleic acid signal. The two strategies based on the branched DNA nanostructure would effectively improve the sensitivity for Point-of-Care testing. The platform shows great promise in rapid nucleic acid detection with excellent specificity and selectivity.

## 2. Results

### 2.1. Mechanism of the Electrochemical Biosensor for Nucleic Acids Determination

**Figure 1:**
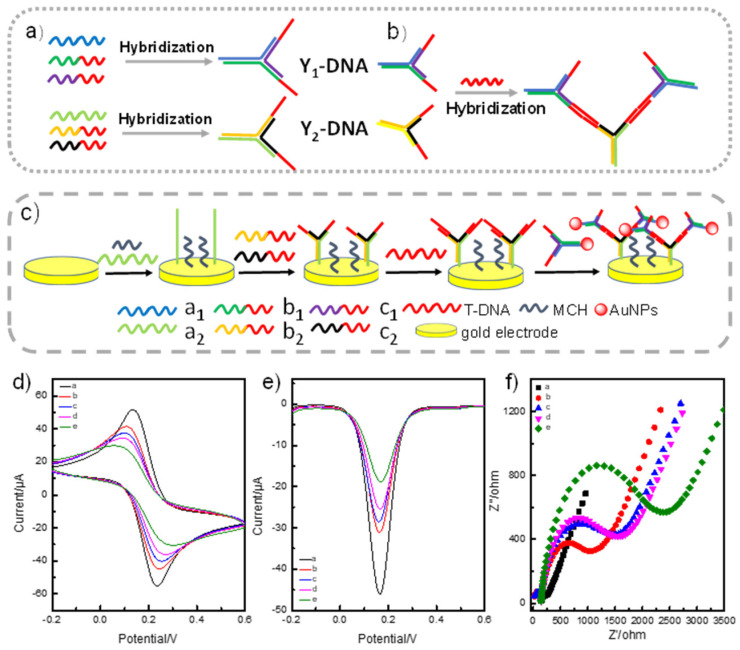
(**a**) The schematic illustration for branched DNA (Y_1_ and Y_2_-DNA) synthesis. Y-DNA was prepared by annealing three ssDNA. (**b**) The hybridization between probe and amplifier triggered by T-DNA. (**c**) The assay platform based on branched DNA and AuNPs-branched DNA. (**d**) Cyclic voltammetry responses, (**e**) DPV response, and (**f**) Nyquist plots. The solid lines a, b, c, d, and e in (**d**–**f**) represented the signal from bare gold electrode, Y_1_-DNA-modified gold electrode, Y_1_-DNA-modified gold electrode, and the detection of T-DNA, amplifier on the Y_2_-DNA modified gold electrode in 0.1 M KCl solution containing 5 mM [Fe(CN)_6_]^3−/4−^].

Our detection system involved two essential parts to effectively improve the sensitivity of specific nucleic acid detection: Branched DNA (Y_1_-DNA)-based target DNA (T-DNA) recognition and DNA (Y_2_-DNA)-modified AuNPs-based signal amplification (Figure 1a,b). As illustrated in Figure 1a, we initially used three single-stranded DNA to synthesize Y_1_/Y_2_-DNA as a probe with three sticky ends, respectively (Table S1). Y_1_-DNA with multiple sticky ends was designed and prepared for T-DNA recognition. One sticky end of Y_1_-DNA with the thiol group (-SH) was first assembled on the gold electrode through the Au-S bond. The other two sticky ends of Y_1_-DNA were employed to capture and recognize T-DNA via hybridization. In addition, Y_2_-DNA-modified AuNPs were synthesized as an amplifier to improve the sensitivity. From Figure 1b,c, we can see that Y_1_ and Y_2_ DNA contained different sticky ends, and the sticky ends of Y_1_-DNA and Y_2_-DNA were complementary with the 3′ end and the 5′ end of T-DNA, respectively (Figure 1b). Through this special sequence design, T-DNA acted as a linker, hybridizing with the complementary sticky ends of Y_1_-DNA and Y_2_-DNA, leading to the formation of a DNA sandwich nanostructure for the detection system. To further improve the sensitivity, we chose AuNPs as an amplifier to increase the T-DNA electronic signal. Therefore, partial sequences of T-DNA can also hybridize with Y_2_-DNA-modified AuNPs. In brief, T-DNA participated in the two reactions simultaneously. Partial sequences of T-DNA hybridized with Y_1_-DNA and other partial sequences linked to Y_2_-DNA-modified AuNPs. In the detection process, T-DNA was recognized and linked to Y_1_-DNA on the surface of the electrode, and it was further linked to the amplifier of AuNPs-DNA to increase the signal. The two reactions produced signal changes to achieve T-DNA detections. (Figure 1c). The hybridization recognition processes of hybridization between Y_1_-DNA and T-DNA altered the surface structure of the electrode, which affected the electro active surface area and electron transfer [Fe(CN)_6_]^3−/4−^. Thus, the recognition process of T-DNA induced a peak current change (Figure 1d). The redox reaction for Fe^2+/3+^, which was seen as an electronic medium, could easily occur on the electrode surface. The resistance of the electrode surface was enhanced owing to the non-conductivity of the complexes when binding of the target DNA-induced DNA nanostructures changed their structure. This impeded the proton transfer of iron ions on the electrode surface, leading to a decrease in the peak current. The DPV voltammogram signal decreased when the T-DNA and the amplifier were added, which further proved the mechanism (Figure 1e). Meanwhile, the resistance of the electrode increased with the T-DNA and the amplifier. These results also agreed with the CV and DPV values of the electrodes (Figure 1f). All the above results verified the successful preparation of the biosensor.

### 2.2. Characterization of the Branched-DNA-Based Sensor

**Figure 2:**
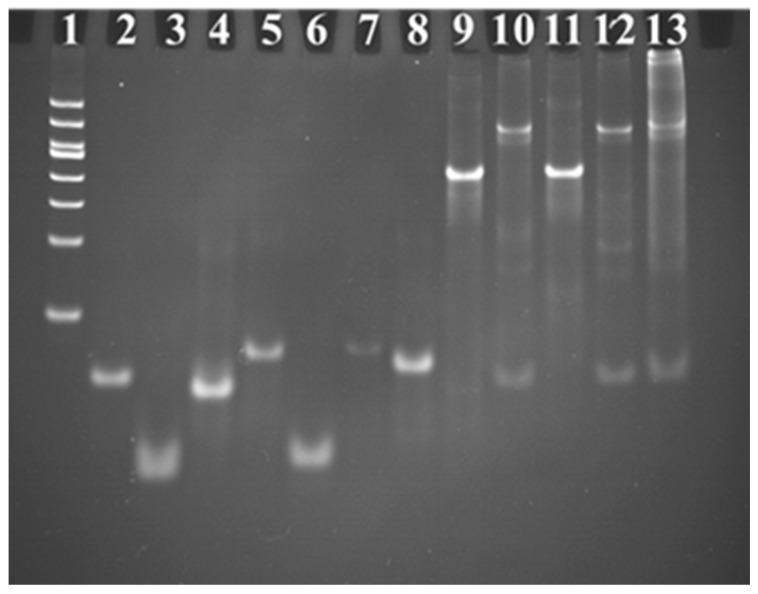
Confirmation of branched DNA formation by 12% polyacrylamide gel electrophoresis analysis. Gel electrophoresis analysis of ladder, target DNA, ssDNA_a1_, ssDNA_b1_, ssDNA_c1_, ssDNA_a2_, ssDNA_b2_, ssDNA_c2_, Y_1_-DNA, Y_1_-DNA + T-DNA, Y_2_-DNA, Y_2_-DNA + T-DNA, and Y_1_-DNA + T-DNA + Y_2_-DNA from Lanes 1 to 13, respectively. The concentrations of ssDNA were all 1 M.

Since the branched DNA represent the key point for the fabrication of the sensor, polyacrylamide gel electrophoresis was first performed to validate the successful synthesis and structure of Y_1_-DNA and Y_2_-DNA. The mixture of three oligo DNAs was heated to 90 °C for 5 min and then allowed to cool on ice for 15 min, subsequently placed for 20 min at room temperature to form Y_1_-DNA and Y_2_-DNA. Then the products were injected into gel electrophoresis. Figure 2 shows that the Y_1_ and Y_2_-DNA band was apparently retarded compared with the ssDNA bands (ssDNA_a1_, ssDNA_b1_, ssDNA_c1_, ssDNA_a2_, ssDNA_b2_, and ssDNA_c2_). Since Y-DNA with a larger size and higher molecular weight theoretically moved slower than the small fragments of ssDNA, these results demonstrated that Y_1_-DNA and Y_2_-DNA were successfully synthesized. Meanwhile, the result also illustrated the hybridization of Y_1_-DNA, Y_2_-DNA, and T-DNA. The high molecular weight of hybridization between Y_1_-DNA, Y_2_-DNA, and T-DNA was closer to the gel hole than other samples. It demonstrated that T-DNA could effectively link with Y_1_-DNA and Y_2_-DNA.

**Figure 3:**
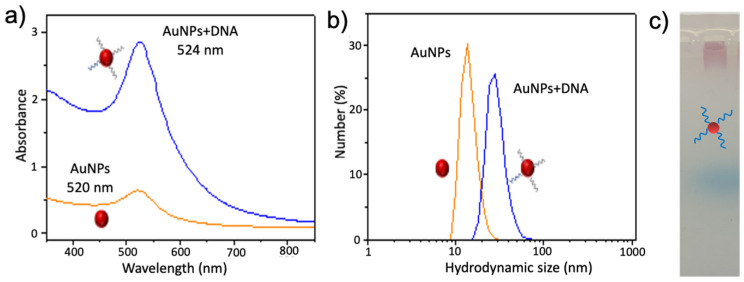
(**a**) The UV-vis spectrum, (**b**) dynamic Light Scattering (DLS), and (**c**) gel electrophoresis analysis of AuNPs and DNA-modified AuNPs. The red circles presented AuNPs.

In our detection system, Y_1_-DNA and Y_2_-DNA-modified AuNPs were used as the probe and amplifier to detect T-DNA, respectively. To effectively increase the sensitivity, Y_2_-DNA was modified using AuNPs to further enhance the detection signal of T-DNA. We first synthesized and characterized the Y_2_-DNA-modified AuNPs by UV-vis, dynamic light scattering (DLS), and electrophoresis (Figure 3). As can be seen in Figure 3a, the absorbance of AuNPs shifted from 520 nm to 524 nm after the modification of Y_2_-DNA, indicating that Y_2_-DNA was successfully attached to the surface of AuNPs. Then, dynamic light scattering (DLS) analysis was carried out to evaluate the diameter of AuNPs and DNA-modified AuNPs. The diameter of DNA-AuNPs was 50 nm, which was much larger than that of the bare AuNPs (12 nm) (Figure 3b). In addition, the gel electrophoresis analysis was further performed to investigate the DNA-AuNPs. AuNPs did not move in agarose gel, while DNA-modified AuNPs could move, owing to the link between DNA and AuNPs (Figure 3c). It also proved that DNA had been linked with AuNPs. All the above results show that the DNA-modified AuNPs were successfully prepared.

### 2.3. Optimization of the Assembly of Branched DNA Hybridization

In our system, the hybridization reaction played an important role in the synthesis of the probe, amplifier, and target nucleic acid detection. Meanwhile, the sensitivity of the detection platform also depended on the hybridization between T-DNA and Y-DNA. Therefore, the effects of various conditions on the assembly of Y_1_-DNA and Y_2_-DNA were systematically evaluated, including different NaCl concentrations, hybridization times, temperatures, and pH. As illustrated in Figures S1 and S2, a concentration of 0 to 120 mM effectively synthesized Y_1_-DNA and Y_2_-DNA, respectively. Further, the synthesized reaction time required was only 5 min. In addition, there was a wide range of pH from 5 to 11, excluding pH lower than 3. The result showed that Y_1_-DNA and Y_2_-DNA could be prepared in various temperatures from 4 °C to 85 °C. Figures S1 and S2 results demonstrated that the probe (Y_1_-DNA) and amplifier (Y_2_-DNA) could be easily and conveniently synthesized in a moderate environment.

**Figure 4:**
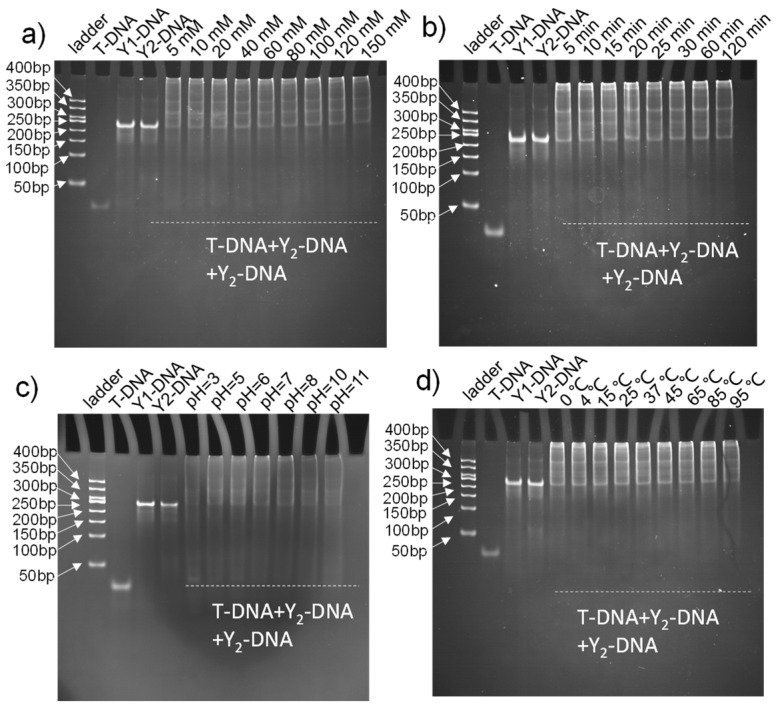
Polyacrylamide gel electrophoresis analysis of hybridization reaction of Y_1_-DNA and Y_2_-DNA by T-DNA triggered under different conditions of NaCl concentration (**a**), reaction time (**b**), pH (**c**), and temperature (**d**).

In addition, the hybridization conditions between T-DNA and Y_1_-DNA/Y_2_-DNA were also optimized in detail. The hybridization molecules of T-DNA and Y-DNA remained in the electrophoresis gel hole with smears in the lane, demonstrating the successful formation of assembly DNA, which had higher molecular weight than Y-DNA under different conditions (Figure 4), indicating the effectiveness of recognition and amplification in different conditions, excluding pH lower than 3. We found that the hybridization reactions were completed in a very short time (5 min), indicating that recognition and amplification could be finished in a short period of time.

### 2.4. Hybridization Sensitivity for T-DNA by the Branched DNA-Based Electrochemical Platform

**Figure 5:**
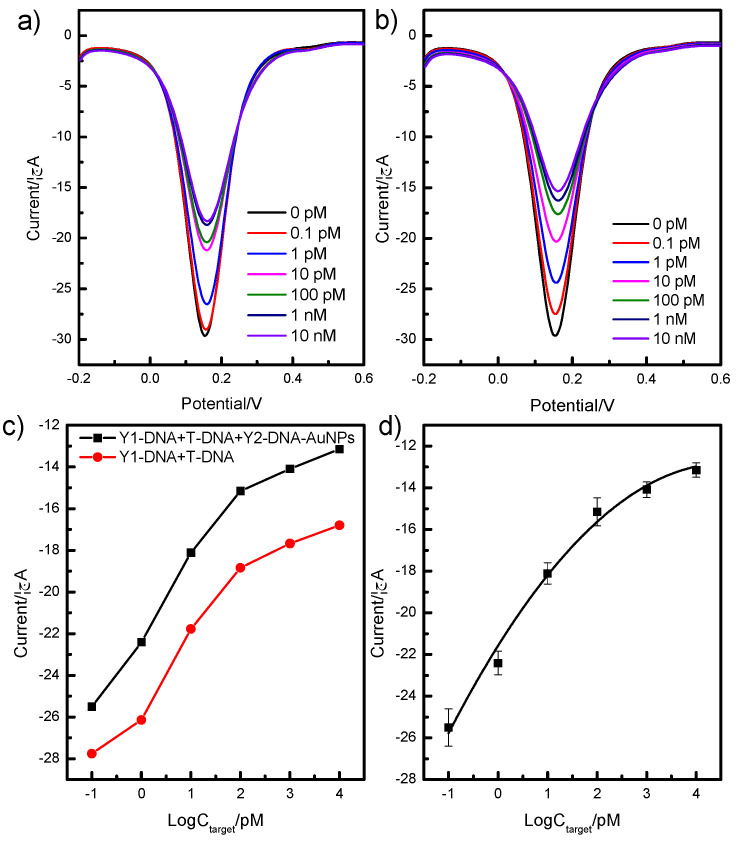
The analytical performance of the detection system based on Y_1_-DNA and Y_1_-DNA/Y_2_-DNA-modified AuNPs. Sensitivity of DPV response for Y_1_-DNA (**a**) and Y_1_-DNA/Y_2_-DNA-modified AuNPs (**b**) incubated with different concentrations of T-DNA from 0.1 pM to 10 nM and the relationship between the peak currents and the concentrations of T-DNA (**c**), (**d**) the linear relationship between the peak currents and the concentration target.

To examine the sensitivity of a branched-DNA-based sensor, a series of different concentrations of T-DNA were detected via the above detection strategy. Firstly, quantitative analysis based on the Y_1_-DNA probe was performed using DPV under the experimental conditions (pH = 8.0, incubation time was 15 min) to validate the T-DNA detection. The electrochemical sensor was incubated in PBS solutions with various T-DNA concentrations for 15 min (Figure 5a). With T-DNA concentrations increasing from 0.1 pM to 10 nM, the peak current values decreased accordingly. The results indicated that T-DNA was captured by the Y_1_-DNA on the surface of the electronic chip, and the electron transition between the electrode and the solution was impeded.

Moreover, we further evaluated the sensitivity of the detection platform using Y_2_-DNA-modified AuNPs (Figure 5b). In comparison with only the Y_1_-DNA probe, the frequency shifts of the combination method of Y_1_-DNA and Y_2_-DNA-modified AuNPs as an amplifier were dramatically enhanced (Figure 5c), which indicates that the amplifier could effectively increase the sensitivity of the detection platform for T-DNA. The curvilinear relationship between the peak current and the logarithm of the T-DNA concentration was obtained with a range from 0.10 pM to 10 nM and a correlation coefficient of 0.987. The equation was Y = −21.57 + 3.79 logC − 0.41 logC^2^. The limit of detection was 0.09 pM (S/N = 3).

### 2.5. Selectivity and Recovery Test of the Electrochemical Sensor

**Figure 6:**
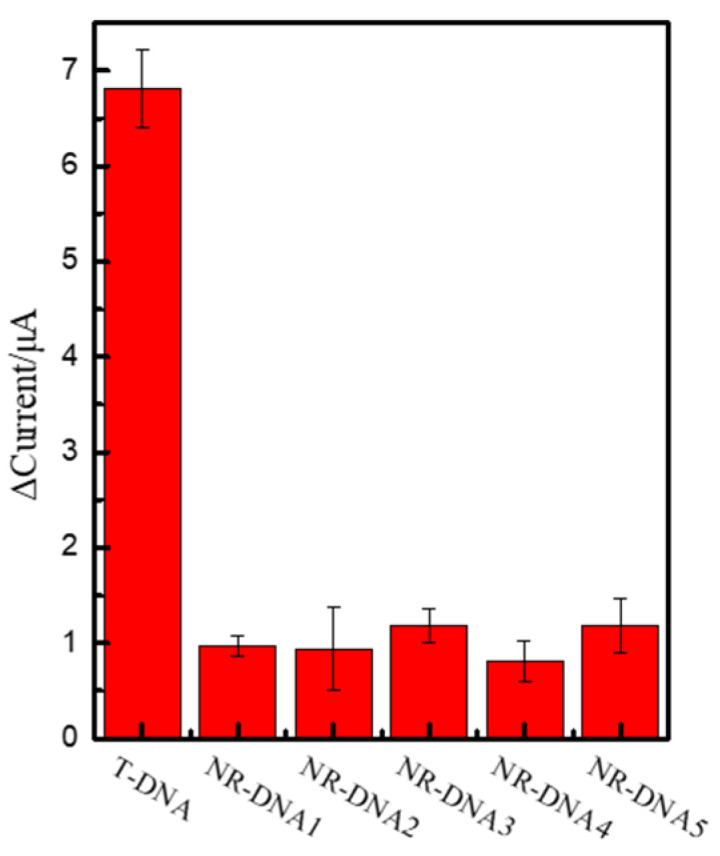
Selectivity of the detection platform; DPV responses of the probe in the presence of interferent DNAs.

Owing to the nonspecific adsorption between the probe and interferents in complicated samples, the interferents tended to influence the test results and even cause false positives. Therefore, selectivity is a critical parameter for evaluating the accuracy of practical sample detection. Therefore, we selected five single-stranded DNA with similar structures (non-relevant DNA, NR-DNA) as interferents to test the selectivity of the sensors. The concentrations of T-DNA, NR-DNA_1_, NR-DNA_2_, NR-DNA_3_, NR-DNA_4_, and NR-DNA_5_ were all 0.1 μM. An appreciable signal was observed only when T-DNA was added to the detection system, whereas no obvious changes were observed when other interfering DNA strands were added (Figure 6). The results indicated that the detection platform was highly selective to T-DNA. The results demonstrated that the NR-DNA samples had no effect on the frequency of the electrochemical biosensor. However, T-DNA significantly decreased the frequency signal, and it indicated that the detection platform had great specificity for T-DNA.

In order to test the accuracy of the branched DNA detection system for the real samples, we prepared simulated samples to validate the recovery of the detection platform. Simulated samples included NR-DNA and T-DNA. As shown in Table 1, the recovery for T-DNA was in a range of 91.8% to 97.3%, which was within the acceptable range. The results demonstrated that the constructed biosensing system for T-DNA detection was feasible for application in specific signal strand DNA detection with high accuracy.

## 3. Materials and Methods

### 3.1. Reagents and Materials

All DNA oligonucleotides were synthesized and purified by Jinweizhi Biotechnology Co., Ltd. (Tianjin, China), and their base sequences are listed in Table S1. 6-Mercaptan-1-hexanol (MCH) was purchased from Aladdin (Shanghai, China). Agarose, Tris (2-carboxyethyl) hydrochloride (TCEP), TAE buffer, TBE buffer and Tris (hydroxymethyl) aminomethane (Tris-HCl) were purchased from Beijing Solarbio (Beijing, China). The piranha solution comprised 98% H_2_SO_4_ and 30% H_2_O_2_. The ultrapure water was obtained using the UPH water purification system (18.2 MΩ, UPH-I).

All electrochemical measurements were performed on a CHI660E electrochemical workstation (Shanghai Chenhua Instruments, Shanghai, China) with a conventional three-electrode system composed of platinum wire as an auxiliary, a saturated calomel electrode as a reference, and a 2-mm-diameter gold electrode (GE) as a working electrode. Agarose gel electrophoresis and polyacrylamide gel electrophoresis (PAGE) experiments were performed on the electrophoresis apparatus (Beijing Liuyi, Beijing, China). Gel images were recorded on an imaging system (JY04S-3C). Centrifugation was performed by a centrifuge (Eppendorf, Germany).

### 3.2. Synthesis of Branched DNA and DNA Modified AuNPs

Branched DNA (Y_1_ DNA) structures were synthesized by mixing the same molar amount of oligonucleotide strands, ssDNA_a1_, ssDNA_b1_, and ssDNA_c1_, in the 80 mM NaCl solution [25]. Using the above method, we also used ssDNA_a2_, ssDNA_b2_, and ssDNA_c2_ to synthesize branched DNA (Y_2_ DNA), which was utilized to modify the AuNPs as an amplifier.

AuNPs were prepared according to the literature [26]. Briefly, 10 mL of 38.8 mM trisodium citrate was added to 100 mL of a boiling 1 mM HAuCl_4_ solution and the color of the solution changed from light yellow to deep red. After boiling for 20 min with constant rapid stirring, the AuNPs solution was cooled to room temperature and stored at 4 °C. After that, Y_2_ DNA was added to 3 mL of the AuNPs solution to produce DNA-modified AuNPs. The mixtures were incubated at room temperature for 16 h. It was “aged” in Tris buffer solution (pH = 8.2) and 1 M NaCl for another 24 h. Finally, the solution was centrifuged twice at 9000 rpm for 40 min to remove free DNA. The red precipitates were dispersed in Tris buffer.

### 3.3. Electrode Pretreatment

The gold electrode was soaked in the piranha solution for 10 min and then thoroughly rinsed with ultrapure water to remove other substances. Then, the pretreated electrode was immersed in 0.5 mL of the 0.1 μM thiolated capture probe (a1-DNA) solution for 12 h at 4 °C. After the electrode was rinsed and immersed in 0.5 mL of 1 mM MCH solution for 1 h to block the non-specific binding site at room temperature, the electrochemical biosensor was rinsed with ultrapure water and used for the following operation.

### 3.4. Target DNA Detection by the Electrochemical Biosensor

The Y_1_-DNA with the –SH group was first modified with electrodes to capture the target DNA via hybridization between the end point of branched DNA and half of the target DNA. Further, another half of the target DNA was hybridized with DNA-modified AuNPs. All the hybridization reactions occurred in the 80 mM NaCl solution. Electrochemical detection was performed in a potassium ferricyanide solution, which contained 5 mM Fe (CN)_6_^3−/4−^ and 0.1 M KCl.

### 3.5. Gel Analysis of the Hybridization Reaction

The hybridization reaction was verified by 12% native polyacrylamide gel electrophoresis (PAGE) in 1× TBE buffer and a 3% agarose gel electrophoresis analysis of the conditional optimization in 1× TAE buffer.

## 4. Conclusions

In conclusion, novel branched DNA-electrochemical sensors were designed to successfully detect nucleic acids with DNA-modified AuNPs as an amplifier without thermal cycles and enzymes. The platform could rapidly and sensitively detect specific nucleic acids at the same temperature. In this system, the branched DNA_1_ used as probes had multiple sticky ends to effectively improve the sensitivity of target nucleic acids, while, DNA-modified AuNPs used as an amplifier could further increase the detection signal of target DNA. Under optimum conditions, the limit of detection of this detection platform could go as low as 0.09 pM. The correlation curve relationship was presented between the peak current and the logarithm of target DNA concentration, which ranged from 0.10 pM to 10 nM. Additionally, the electrochemical detection platform also exhibited excellent selectivity with an expeditious response. Owing to the rapid reaction, simple operation, and enzyme-free nature, the detection platform shows great promise in applications such as genetic diseases, clinical molecular diagnostics, and forensic identification.

## Figures and Tables

**Table 1:** Recoveries of electrochemical detection system for specific nucleic acids (n = 3).

Sample	Add DNA	Found DNA	Recovery (%)
1	5 pM	4.59 pM	91.8
2	50 pM	48.63 pM	97.3
3	500 pM	486.26 pM	97.3

## Data Availability

Not applicable.

## Supplementary Materials

The following supporting information can be downloaded at: https://www.mdpi.com/article/10.3390/ijms241612565/s1.
