# Supplementary material for: Branched DNA-Based Electrochemical Biosensor for Sensitive Nucleic Acids Analysis with Gold Nanoparticles as Amplifier
Source: Int J Mol Sci. 2023 Aug 8;24(16):12565. doi: 10.3390/ijms241612565 (PMC10454004; doi:10.3390/ijms241612565)
Supplement: Supplementary file 1 [file ijms-24-12565-s001.zip › ijms-2521734-supplementary.pdf]

Branched DNA-based electrochemical biosensor for sensitive nucleic acids analysis with gold nanoparticles as amplifier

Zhikun Zhang*, Chunyan Shang, Cuixia Hu, Yumin Liu, Jilong Han*

School of Chemical and Pharmaceutical Engineering, Hebei University of Science and Technology, Shijiazhuang, 050018, China

*Corresponding author E-mail address. zhikun.zhang@hebust.edu.cn and hanjilong@hebust.edu.cn

Table S1. Oligouncleotide sequences were employed to synthesize branched DNA or partcipate in hybridization chain reaction.

Strand	Sequence (5’-3’)	Use
T-DNA	CGACATGTCATAGCTTATCAGACTGATGTTGACGATTCTCTA	Target DNA
a_1-DNA	GAAGCTGCCAGTACCAATCCTGTCGCACAAAAAAAAAAAA-SH	
b_1-DNA	GGAGACTAGATCATGTACTGGCAGCTTCTAGAGAATCGTCAACATCAGT	Y_1-DNA Probe
c_1-DNA	GTGCGACAGGATTGATGATCTAGTCTCCTAGAGAATCGTCAACATCAGT	
a_2-DNA	SH-AAAAAAAAAAAACACGCTGTCCTAACCATGACCGTCGAAG	Amplifer
b_2-DNA	CTGATAAGCTATGACATGTCGCTTCGACGGTCATGTACTAGATCAGAGG	Y_2-DNA
c_3-DNA	CTGATAAGCTATGACATGTCGCCTCTGATCTAGTAGTTAGGACAGCGTG	

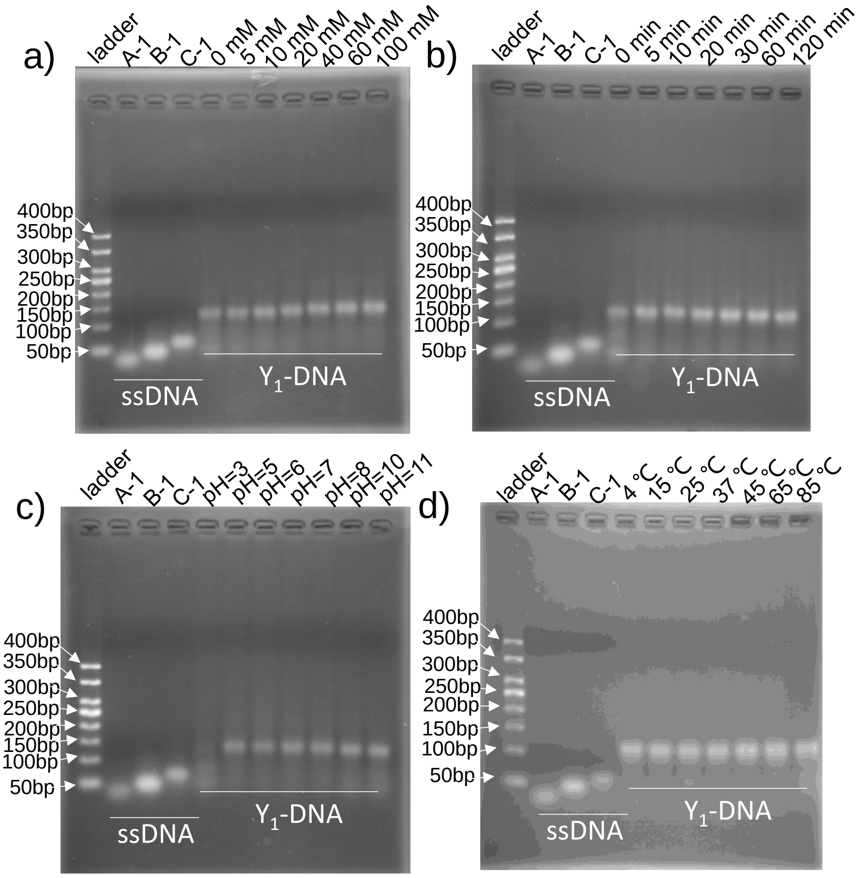

Figure S1. Gel electrophoresis analysis (3%) of Y_1-DNA prepared under different conditions. The Y_1-DNA was prepared in presence of various NaCl concentrations (a), incubation time (b), pH (c) and temperature (d).

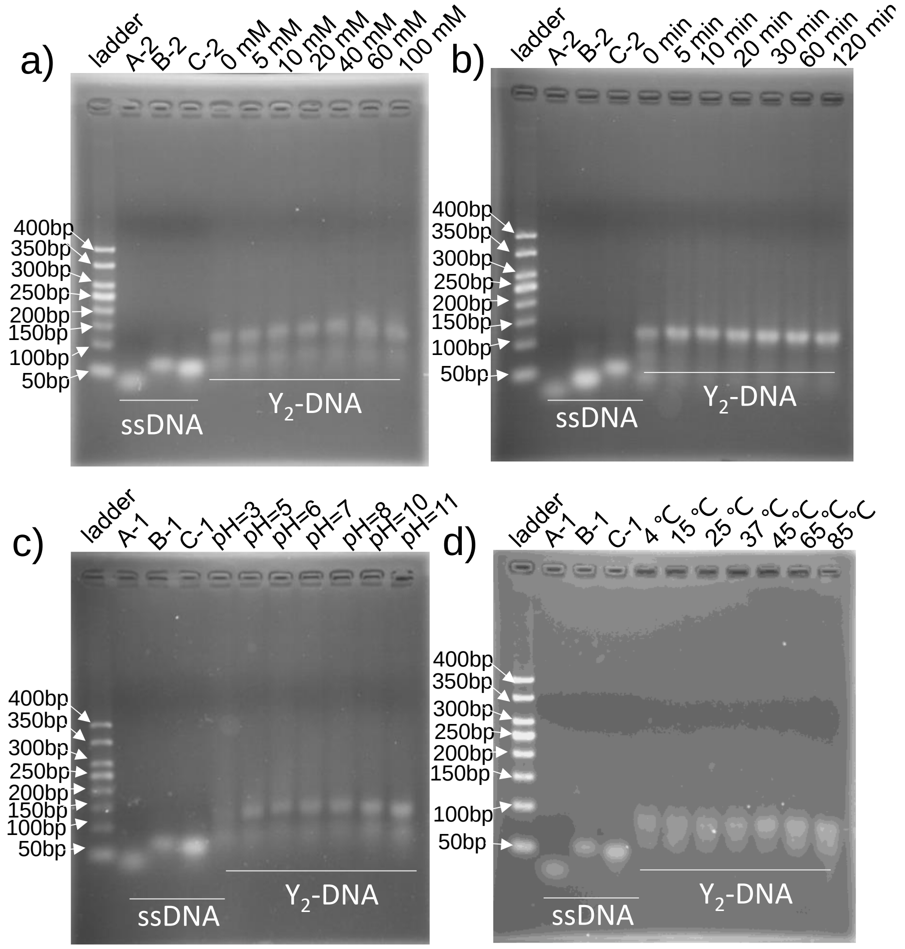

Figure S2. Gel electrophoresis analysis (3%) of Y_2-DNA prepared under different conditions. The Y_2-DNA was prepared in presence of various NaCl concentrations (a), incubation time (b), pH (c) and temperature (d).
